# An algorithm for the reduction of genome-scale metabolic network models to meaningful core models

**DOI:** 10.1186/s12918-015-0191-x

**Published:** 2015-08-19

**Authors:** Philipp Erdrich, Ralf Steuer, Steffen Klamt

**Affiliations:** Max Planck Institute for Dynamics of Complex Technical Systems, Sandtorstrasse 1, Magdeburg,, D-39106 Germany; Humboldt University of Berlin, Institute for Theoretical Biology, Invalidenstrasse 43, Berlin, D-10115 Germany

**Keywords:** Constraint-based modeling, Model reduction, Stoichiometric models, *Escherichia coli*

## Abstract

**Background:**

Constraint-based analysis of genome-scale metabolic models has become a key methodology to gain insights into functions, capabilities, and properties of cellular metabolism. Since their inception, the size and complexity of genome-scale metabolic reconstructions has significantly increased, with a concomitant increase in computational effort required for their analysis. Many stoichiometric methods cannot be applied to large networks comprising several thousand reactions. Furthermore, basic principles of an organism’s metabolism can sometimes be easier studied in smaller models focusing on central metabolism. Therefore, an automated and unbiased reduction procedure delivering meaningful core networks from well-curated genome-scale reconstructions is highly desirable.

**Results:**

Here we present *NetworkReducer*, a new algorithm for an automated reduction of metabolic reconstructions to obtain smaller models capturing the central metabolism or other metabolic modules of interest. The algorithm takes as input a network model and a list of protected elements and functions (phenotypes) and applies a pruning step followed by an optional compression step. Network pruning removes elements of the network that are dispensable for the protected functions and delivers a subnetwork of the full system. Loss-free network compression further reduces the network size but not the complexity (dimension) of the solution space. As a proof of concept, we applied *NetworkReducer* to the *i*AF1260 genome-scale model of *Escherichia coli* (2384 reactions, 1669 internal metabolites) to obtain a reduced model that (i) allows the same maximal growth rates under aerobic and anaerobic conditions as in the full model, and (ii) preserves a protected set of reactions representing the central carbon metabolism. The reduced representation comprises 85 metabolites and 105 reactions which we compare to a manually derived *E. coli* core model. As one particular strength of our approach, *NetworkReducer* derives a condensed biomass synthesis reaction that is consistent with the full genome-scale model. In a second case study, we reduced a genome-scale model of the cyanobacterium *Synechocystis* sp. PCC 6803 to obtain a small metabolic module comprising photosynthetic core reactions and the Calvin-Benson cycle allowing synthesis of both biomass and a biofuel (ethanol).

**Conclusion:**

Although only genome-scale models provide a complete description of an organism’s metabolic capabilities, an unbiased stoichiometric reduction of large-scale metabolic models is highly useful. We are confident that the *NetworkReducer* algorithm provides a valuable tool for the application of computationally expensive analyses, for educational purposes, as well to identify core models for kinetic modeling and isotopic tracer experiments.

**Electronic supplementary material:**

The online version of this article (doi:10.1186/s12918-015-0191-x) contains supplementary material, which is available to authorized users.

## Background

Stoichiometric and constraint-based analysis of metabolic networks has become a key methodology to gain insights into functions, capabilities and properties of cellular metabolism [1–3]. Applications of metabolic network modeling include, for example, (i) simulation and prediction of metabolic phenotypes (metabolic flux distributions) for cellular growth under certain environmental conditions, either in the wild-type or in mutants containing certain gene knockouts; (ii) the identification of maximal growth or product yields and the respective metabolic pathways leading to optimal yields; (iii) the computation of intervention strategies increasing the synthesis of certain products (metabolic engineering); or (iv) analysis of general structural properties such as coupled reactions or the identification of gaps in reconstructed metabolic networks. Until the end of the 90’s, stoichiometric models had usually not more than 100 (central metabolic) reactions. With the advent of annotated genomes, however, larger metabolic models were constructed for different organisms and the first reconstruction reached genome-scale level at the turn of millennium. Today, more than 100 genome-scale metabolic network reconstructions for diverse organisms from all kingdoms of life have been published and examined [4]. These models are still evolving with new biological knowledge, as can be exemplified by network reconstructions for *Escherichia coli* (*E. coli*). The first *E. coli* genome-scale model (*i*JE660) was published in 2000 [5] and consisted of 627 reactions and 438 metabolites. It was updated by Reed et al. in 2003 [6] leading to an expanded network with 931 reactions and 625 metabolites (*i*JR904). The next iteration (*i*AF1260) was published in 2007 [7] and contained as many as 2077 reactions and 1039 metabolites. The latest update was made in 2011 (*i*JO1366 [8]) and comprises 2251 reactions and 1136 metabolites. Numerous applications of these models demonstrated the power of constraint-based modeling in general and gained invaluable insights on the metabolism of *E. coli* [2].

With increasing size and complexity of genome-scale models the computational effort for their analysis increased as well. Some stoichiometric methods, for example those that require enumeration of elementary modes [9], cannot be applied to networks consisting of several thousand reactions due to computational intractability. Furthermore, metabolic flux analysis can usually only be used with models of the central metabolism since intracellular fluxes would poorly be determined in the full system [10], even if data from isotopic tracer experiments are available [11]. Similar arguments hold for kinetic modeling, which, due to lacking knowledge about kinetic mechanisms and parameters, can usually only be applied to smaller modules of the complete metabolic network. Finally, metabolic core models may sometimes be more suitable to study and understand basic principles of an organism’s (central) metabolism. In all these cases, it would be most desirable to take a well-curated genome-scale model and to reduce this model to a certain core or module while keeping key elements or/and important functional properties. While some specific examples for network reduction of genome-scale models have been described in the literature [10, 12, 13], we are not aware of an automated and flexible network reduction approach that can generically be applied to any metabolic network. In this work we present such an algorithm and demonstrate its applicability and power by reducing a genome-scale metabolic network of *E. coli* (*i*AF1260) to a meaningful core network representing the central metabolism. In a second case study, we reduce a genome-scale model of a cyanobacterial species to a small metabolic module which comprises photosynthetic core reactions and the Calvin-Benson cycle and allows synthesis of both biomass and a biofuel (ethanol).

## Methods

### Stoichiometric networks and constraint-based modeling

Metabolic network models with *m* internal metabolites and *n* reactions can be represented by a *m × n* stoichiometric matrix **N**. The basic assumption of steady state (internal metabolite concentrations do not change) leads to the metabolite balancing equation

1 $$ \mathbf{N}\mathbf{r}=\mathbf{0} $$

where **r** is the vector of net reaction rates (also called flux or rate vector). The solutions **r** satisfying (1) form the null space of **N** whose dimension is given by the degrees of freedom (*dof*) defined as

2 $$ dof=n- rank\left(\mathbf{N}\right). $$

Information on reaction rates (e.g., reversibilities and maximal flux capacities) can be integrated by setting lower and upper boundaries for some reaction rates:

3 $$ {\alpha}_i\le {r}_i\le {\beta}_i. $$

The set of flux vectors **r** fulfilling (1) and (3) forms, in general, a polyhedron which can be bounded or unbounded. Flux balance analysis (FBA [14]) is often used to find optimal flux vectors in this polyhedron by maximizing a specific linear objective function

4 $$ \underset{\mathbf{r}}{maximize}\kern0.6em z={\mathbf{c}}^T\mathbf{r}. $$

Typical examples are maximization of biomass production or of formation of a certain product. While the optimal value *z* in (4) is always unique, infinite optimal flux vectors **r** achieving the maximal *z* may exist (especially in genome-scale networks). Here, flux variability analysis (FVA; [15]) can be used to identify the feasible flux ranges in the network by determining for each reaction the minimum and the maximum reaction rate under the given constraints (1) and (3).

### Network reduction algorithm

Our network reduction algorithm *NetworkReducer* reduces a given large-scale metabolic network to a smaller subnetwork thereby keeping desired features of the full network. *NetworkReducer* accepts the following specifications of protected parts, properties, and phenotypes (specific cellular behaviors, functions, capabilities):

- Set of protected metabolites *P*^*M*^: all metabolites in *P*^*M*^ must be retained in the reduced network.
- Set of protected reactions *P*^*R*^: all reactions in *P*^*R*^ must be retained in the reduced network. Optionally, one may also specify whether protected reactions must be feasible (i.e., whether for each protected reaction *i* ∈ *P*^*R*^ at least one flux vector r must exist such that *r*_*i*_ ≠ 0).
- Protected functions and phenotypes: described by appropriate inequalities (see below).
- Minimum degrees of freedom: the *dof* (equation (2)) of the reduced network may not fall below a minimum threshold: *dof* ≥ *dof*_*min*_.
- Minimum number of reactions (*n* ≥ *n*_*min*_).

Specifications of type (a) and (b) allow protection of certain reactions and metabolites which will not be deleted by the algorithm. A typical scenario is to preserve reactions and metabolites from the central metabolism of a genome-scale network. For protected metabolites we demand that at least one (non-blocked) reaction involving this metabolite must be maintained in the network.

A key feature of our algorithm is the consideration of desired (protected) functions and phenotypes (specifications of type (c)). Each protected functionality *k* is described by a corresponding set of linear equalities/inequalities (*s* denotes the number of protected functions and phenotypes; the terms “phenotypes” and “functions” are used interchangeably in the following):

5 $$ {\mathbf{D}}_k\mathbf{r}\le {\mathbf{d}}_k\kern0.75em k=1\dots s. $$

In the *E. coli* case study presented in the Results section, for instance, given a maximal substrate (glucose) uptake rate of 10 mmol/gDW/h and a non-growth associated ATP maintenance (ATPM) requirement of 8.39 mmol/gDW/h [7] (these values are included as flux bounds in (3)), we will demand that the maximal growth rate in the reduced network should be close (99.9 %) to the maximal growth rate (*μ*_*max_full*_) of the full network. Inequalities describing these constraints in the style of (5) are

6 $$ \begin{array}{l}\kern0.24em {r}_{Glc\_ up}\le 10\\ {}\kern0.96em -\mu \le -0.999{\mu}_{max\_ full}\end{array} $$

which can be integrated in an appropriate matrix/vector pair **D**_1_/**d**_1_. We thus demand that at least one steady state flux vector (fulfilling (1) and (3)) must exist in the reduced network that obeys (6) and thus achieves maximal growth rate. In the case study we will additionally demand that under *anaerobic* conditions the maximal (anaerobic) growth rate of the full network (*μ*_*max_full_anaerobic*_) can also be reached by the reduced network:

7 $$ \begin{array}{l}\kern0.24em {r}_{Glc\_ up}\le 10\\ {}\kern0.24em {r}_{O2\_ up}\le 0\\ {}\kern0.96em -\mu \le -0.999{\mu}_{max\_ full\_ anaerobic}.\end{array} $$

Inequalities (7) can be properly described by a second pair **D**_2_/**d**_2_. Hence, **D**_1_/**d**_1_ and **D**_2_/**d**_2_ describe two (independent) functionalities of the full system to be preserved in the reduced network (i.e., for each functionality *k*, one steady-state flux vector **r**_*k*_ must exist in the reduced network fulfilling the respective inequalities). Other phenotypes, for example, the production of a certain compound with high yield, could be protected as well.

**Fig. 1:**
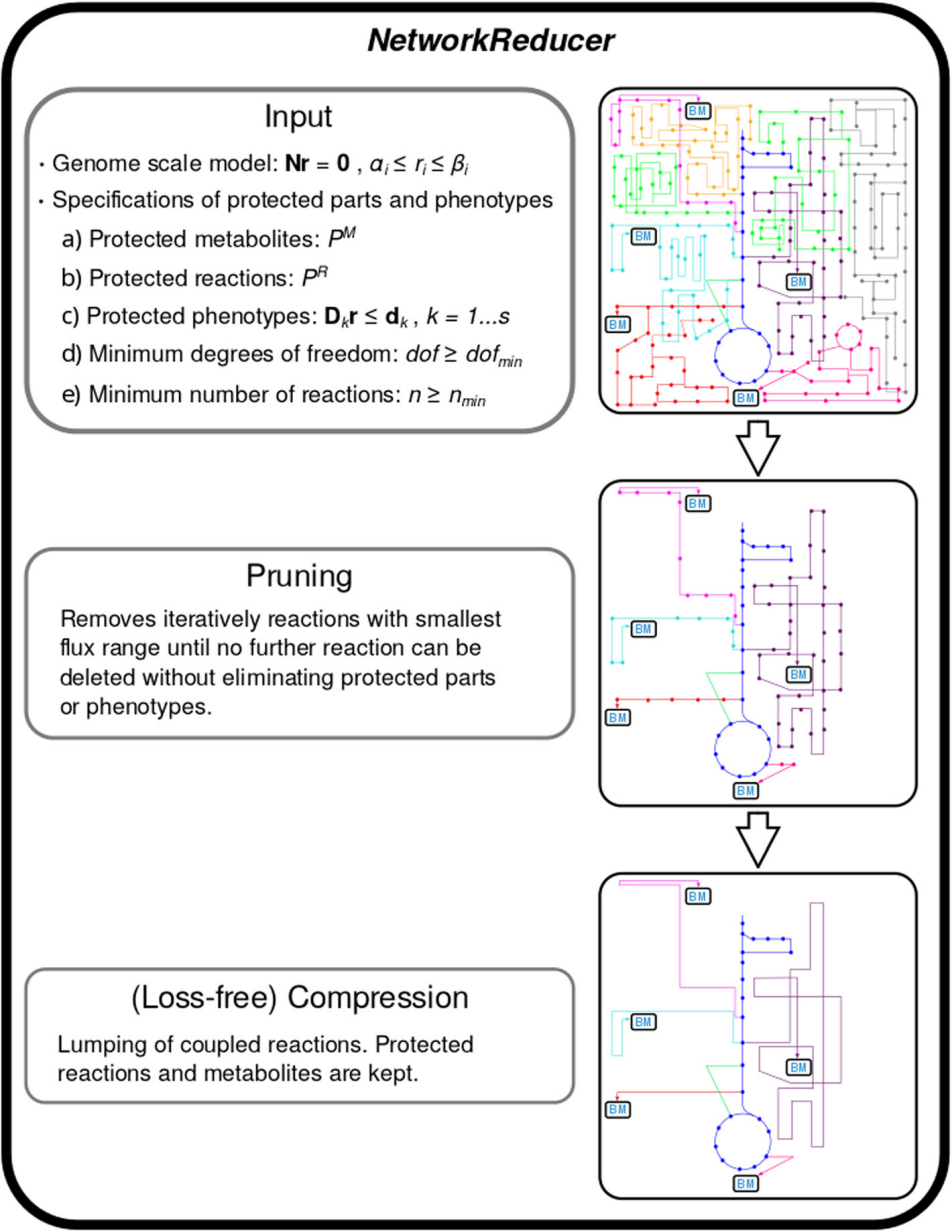
Schematic representation of the *NetworkReducer* algorithm. The figs. on the right-hand side illustrate the result of the step on the left-hand side

The actual network reduction algorithm (see pseudo-code below and Fig. 1) starts with a preprocessing step, which checks the feasibility of the protected functions in the original network and removes (non-protected) blocked reactions. The main algorithm is divided into two major parts: *network pruning* followed by *network compression*. Network pruning involves a loop which removes iteratively non-protected reactions thereby checking that none of the desired properties (a)-(e) is violated. In each iteration, the algorithm applies FVA in the current network to calculate for each *removable* (non-protected) reaction *i* the feasible flux ranges separately for each protected function *k* defined by **D**_*k*_/**d**_*k*_. With *F*_*i*_^*k*^ we identify the flux range of reaction *i* under the protected function *k, k* = 1…*s,* and determine then the union *F*_*i*_ of all these flux ranges: ${F}_i={\displaystyle \underset{k=1}{\overset{s}{\cup }}{F}_i^k}$. (If no protected function was specified then *F*_*i*_ is defined as the full flux range of reaction *i* in the network.) Two important results can be derived from these flux ranges. First, a reaction having an entirely positive or entirely negative flux range *F*_*i*_^*k*^ for any of the desired functionalities *k* is identified as essential reaction and therefore removed from the list of removable reactions. Second, from the remaining removable reactions, the next deletion candidate is identified as the reaction that has the smallest overall flux range *F*_*i*_. We assume that removal of this reaction maintains a high degree of flux variability in the network (other criteria for selecting the next deletion candidate could be used as well). After deletion of a reaction, feasibility of the protected functions (condition (c)), of protected reactions (if enforced in the specification of (b)) and of protected metabolites (at least one reaction that contains the protected metabolite must be feasible) is tested. If any of the conditions is violated, then the last removed reaction is reinserted and marked as non-removable and the reaction with the second smallest overall flux range *F*_*i*_ is considered. Once a reaction has been removed, the next iteration starts and the flux ranges are recalculated. The main loop of network pruning stops when no further reaction can be deleted without violation of any of the specifications (a)-(e). At the end, unconnected metabolites in the reduced network not participating in any of the remaining reactions are removed from the network.

In a post-processing step, loss-free *network compression* can be (optionally) applied using methods as presented in [16, 17]. In particular, reaction or enzyme subsets (e.g., from linear chains of reactions) will be represented as single overall reactions with collapsed stoichiometries. As a special feature of our compression algorithm, protected reactions and metabolites are kept and excluded from compression. One example illustrating the benefit of loss-free network compression is the following (see also Fig. 1 and Results section): If the central metabolism of a genome-scale model was specified as a protected subnetwork and (optimal) growth as a protected function, then, for each compound (e.g., an amino acid) in the biomass synthesis reaction, network pruning will typically keep one optimal pathway and remove all alternate routes from the respective precursor(s) in the central metabolism to this amino acid. Reactions and metabolites along this pathway were not defined as protected but were nevertheless kept in the system to still allow (optimal) growth. Loss-free network compression will compress the linear pathway from the precursor(s) to the amino acid by replacing the stoichiometric coefficient of the amino acid in the biomass synthesis reaction (BSR) with the cumulative stoichiometries of the precursors and cofactors (ATP, NAD(P)H etc.) needed to synthesize the amino acid in the required amounts. For example, assume we have precursor P (which is part of the protected core network) and the three sequential reactions 2 P➔D; D+NADPH➔F; F+ATP➔A for synthesizing the amino acid A. Furthermore, let A be contained with a stoichiometric coefficient of 2 in the BSR (2 A + …. ➔ Biomass). Network compression will remove A from the BSR and add instead P, ATP, and NADPH with coefficients 4, 2, and 2, respectively. The BSR thus effectively changed to 4 P + 2 ATP + 2 NADPH + (0 A) + … ➔ Biomass. Metabolites D, F and A as well as the three reactions can be afterwards removed. Since ATP, NADPH and other metabolites (precursors) appear in several synthesis pathways, their respective stoichiometric coeffcients along all these pathways are summed up to eventually obtain the cumulative stoichiometry of biomass synthesis. We note that network compression leads to a more compact network representation but, in contrast to network pruning conducted in the first step, it neither changes the *dof* nor the potential behaviors of the network.

With the specifications (a)-(e), many relevant scenarios of network reduction can be handled and two typical examples are demonstrated in the Results section. A key property of our method is that the resulting network generates a true subset of the phenotypes of the full network. Furthermore, if only the pruning step is performed (no compression), the obtained network will represent a proper subnetwork of the full network.

The pseudo-code of the whole NetworkReducer procedure is given below. The algorithm has been integrated in our MATLAB toolbox *CellNetAnalyzer* [18]. Files and scripts used in this study (see Results section) can be downloaded from http://www2.mpi-magdeburg.mpg.de/projects/cna/etcdownloads.html.

## Results and discussion

### Reduction of an *E. coli* genome-scale model

This section aims to give a proof-of-principle of our *NetworkReducer* algorithm using a realistic application example. A typical scenario for a network reduction problem is as follows: given a genome-scale metabolic network with several thousand reactions, the goal is to reduce the network to a core network (with about 80–150 user-defined reactions), usually the central metabolism, while preserving the ability of the virtual organism to grow or/and to produce certain metabolites. As source for a genome-scale network we used the *i*AF1260 model of *Escherichia coli* presented by Feist et al. [7] which contains 2382 reactions and 1668 metabolites and is one of the most frequently used metabolic network models. We compare the outcome of our network reduction routine with an *E. coli* core model presented by Orth et al. [12]. The latter model, denoted in the following by *ColiCore*, covers the central metabolism of *E. coli.* It contains 95 reactions and 72 internal metabolites and was manually derived by Orth et al. with *i*AF1260 serving as a starting point. Accordingly, *ColiCore* and *i*AF1260 use identical identifiers for metabolites and reactions.

We considered the network spanned by the reactions contained in the *ColiCore* model as the “target network” to be reached by network reduction of *i*AF1260. Before we could start with the reduction process, some minor adaptations had to be done to make both models consistent. First, *ColiCore* used the fumarate reductase reaction with ubiquinol-8 as redox carrier (instead with menaquinol-8 as used in *i*AF1260) and we added this reaction also to *i*AF1260 to allow protection of this reaction during the reduction. In addition we introduced a metabolite “biomass” in the genome-scale model, integrated it in the biomass synthesis reaction as a product with stoichiometric coefficient of 1 [gram] and added then also a reaction to “export” this biomass compound. This configuration makes it easier to configure biomass synthesis as a protected function and to keep track of the stoichiometric coefficient of the biomass compound during network compression. The final stoichiometric matrix of the genome-scale model (in the following denoted by *ColiGS*) was thus slightly extended to 2384 x 1669. Flux constraints in the *ColiGS* model were specified such that glucose serves as the only carbon substrate.

The *ColiCore* model contains uptake reactions for glucose but also for some other substrates (including some amino acids). Since we aimed to focus on glucose as the sole carbon substrate, we removed all reactions and metabolites from the *ColiCore* model that are involved in uptake of these substrates. Analogously to the *ColiGS* model, we also included a biomass metabolite in the biomass synthesis reaction and a corresponding “biomass export” reaction. With these changes, the final dimension of the *ColiCore* model was 88 reactions and 69 internal metabolites. Key properties of *ColiGS* and *ColiCore* are summarized in Table 1.

**Table 1:** Properties of *E. coli* network models discussed in the text. *ColiGS* and *ColiCore* are slightly modified versions of *i*AF1260 [7] and of the model presented in [12], respectively. All models are available in SBML format at http://www2.mpi-magdeburg.mpg.de/projects/cna/etcdownloads.html

	*E. coli* genome-scale model (*ColiGS)*	*E. coli* pruned model (*ColiPruned*)	*E. coli* pruned and compressed model (*ColiPrunedComp*)	*E. coli* core model of Orth et al. [12] (*ColiCore*)
# reactions	2384	455	105	88
# int. metabolites	1669	438	85	69
# ext. metabolites	305	33	33	17
degrees of freedom	753	26	26	24
# conservation relations	38	9	6	5
# enzyme subsets (# containing reactions)	321 (890)	23 (413)	22 (62)	23 (55)
*μ*_*max*_ (aerobic)	0.9290 h^−1^	0.9288 h^−1^	0.9288 h^−1^	0.8739 h^−1^
*μ*_*max*_ (anaerobic)	0.2309 h^−1^	0.2309 h^−1^	0.2309 h^−1^	0.2117 h^−1^

After these preliminary steps network reduction of the *ColiGS* network could be started. Phenotypes and elements of the genome-scale model to be maintained in the reduced model were specified as follows. All 88 reactions of the *ColiCore* model were marked as protected reactions (*P*^*R*^ ) in the *ColiGS* model and we also demanded that all these reactions should be feasible (unblocked) in the reduced network. Since all metabolites involved in these reactions are implicitly protected as well we did not explicitly specify protected metabolites (*P*^*M*^ = ∅). Concerning protected phenotypes, we demanded that (i) at least 99.9 % of the maximal growth rate (0.9290 h^−1^; Table 1) in the *ColiGS* model under aerobic conditions and (ii) at least 99.9 % of the maximal growth rate in the *ColiGS* model under anaerobic conditions (0.2309 h^−1^) should be reachable in the reduced network (see equations (6) and (7)). As already described in the Methods section, the maximal glucose uptake rate was set to 10 mmol/gDW/h and minimal ATP maintenance demand to 8.39 mmol/gDW/h. Finally, *dof*_*min*_ and *n*_*min*_ were both set to 1, hence, we aimed to reduce the network as far as possible while keeping the protected reactions and phenotypes.



Network reduction without applying network compression in postprocessing (only pruning) yielded a reduced network model (*ColiPruned*) which already decreased the dimension of the genome-scale network from 2384 reactions / 1669 internal metabolites to 455 reactions / 438 internal metabolites. As demanded, the maximal growth rates under aerobic and anaerobic growth in *ColiPruned* are identical (anaerobic) or very close (aerobic) to the respective maximal rates in the genome-scale model. The *dof* of *ColiPruned* (26) is significantly smaller than in *ColiGS* (753) and already close to the *dof* of *ColiCore* (24) indicating a similar complexity of the solution spaces of *ColiPruned* and *ColiCore*. Even though *ColiPruned* still contains more than 400 reactions, the (1,410,332) elementary modes of this network can be fully enumerated.

The structure of the *ColiPruned* network resembles the second network shown in Fig. 1: many redundant (and suboptimal) pathways to biomass components have been removed while the (protected) core subnetwork was retained. As described in the Methods sections, the remaining linear pathways from the central metabolism to the biomass components can be further compressed to single (lumped) reactions without loss of information (i.e., without loss of solutions or phenotypes). Applying the network compression routine (under consideration of the protected reactions) to *ColiPruned* yields the fully reduced network *ColiPrunedComp*, whose structure is now similar to *ColiCore* and resembles the third network shown in Fig. 1. *ColiPrunedComp* (105 reactions) is much smaller compared to *ColiPruned* (455 reactions) while neither the *dof* nor the number of elementary modes and the possible phenotypes changed. The dimension of *ColiPrunedComp* is now very similar to *ColiCore* (see properties in Table 1), yet, there are still some differences which we analyzed in detail.

*ColiPrunedComp* contains 17 more reactions and 16 more internal metabolites than *ColiCore*. A large fraction (15 reactions and 15 metabolites) of these additional elements arises due to a different description of exchange reactions. The *ColiCore* considers two compartments where metabolites are balanced (cytoplasm and extracellular space) plus (implicitly) an environment. Thus, 3 reactions and 3 balanced species are needed to describe the import of a metabolite M (M_environment_ ➔ M_extracellular space_ ➔ M_cytoplasm_) in the *ColiCore* model (analogous for export). In contrast, *ColiGS* contains additionally a periplasm compartment, hence, uptake of an exchange metabolite M involves 4 exchange reactions and 4 species (M_environment_ ➔ M_extracellular space_ ➔ M_periplasm_ ➔ M_cytoplasm_). Since 15 metabolites can be exported/imported to/from the environment in *ColiCore*, 15 additional species and reactions (for the periplasmic space) must be maintained in the *ColiPrunedComp* model; they can also not be compressed because they are surrounded by protected reactions.

Another observation is that the genome-scale network and its derived reduced models allow higher maximal growth rates than the *ColiCore* model for which we identified two reasons. First, different stoichiometries for biomass synthesis may lead to different biomass yields (discussed below). Second, we found that the remaining two additional (non-exchange) reactions in the *ColiGS* model not contained in the *ColiCore* model allow for higher maximal growth rates; these reactions were kept by the pruning algorithm to maintain the protected phenotype of maximal growth in the *ColiPrunedComp* model (in fact, these two reactions explain the higher *dof* in *ColiPrunedComp* compared to *ColiCore*). The first of these two reactions is related to hydrogen production which turns out to be necessary to attain the maximal anaerobic growth rate. The second reaction is related to respiratory pathways. The *ColiGS* model contains two cytochrome oxidases (cytochrome *bd* and cytochrome *bo*_3_ oxidase). Both oxidize ubiquinol but with different stoichiometry for the translocated (pumped) protons:

8 $$ 2\ {H}^{+}+0.5\ {O}_2+{Q}_8{H}_2={H}_2O+2\ {H}_{periplasm}^{+}+{Q}_8 $$

9 $$ 4\ {H}^{+}+0.5\ {O}_2+{Q}_8{H}_2={H}_2O+4\ {H}_{periplasm}^{+}+{Q}_8 $$

Clearly, the second reaction (9) will lead to a higher ATP yield during respiration and therefore allow a higher growth rate under aerobic conditions. In contrast, the *ColiCore* model contains only reaction (8) translocating two protons accompanied with a lower ATP and biomass yield. This reaction (as all reactions from *ColiCore*) was protected when reducing *ColiGS*, however, to allow maximal growth, the second reaction had also to be kept in *ColiPrunedComp*. In fact, integration of reaction (9) in the *ColiCore* model increases the maximal growth rate to 0.9647 h^−1^ which is even higher than the *μ*_*max*_ of *ColiPrunedComp* (and *ColiGS*).

These remaining discrepancies can be attributed to different stoichiometric coefficients in the biomass synthesis reaction (BSR) of *ColiCore* and *ColiPrunedComp*. For the case of *ColiCore*, the stoichiometric coefficients of the precursor metabolites (such as pyruvate, acetyl-CoA, etc.) in the BSR had to be calculated manually from the known requirements of monomers (amino acids, nucleotides, fatty acids, etc.) in the genome-scale BSR. Accordingly, Orth et al. wrote [12]: “*Since most of the subunits of the cellular macromolecules, such as nucleic acids and amino acids, are not present in the core model, they could not be directly accounted for the biomass reaction. The metabolites in the core model that those macromolecular subunits are synthesized from are included instead. These are precursor metabolites. For example, the amino acid L-alanine is synthesized from pyruvate and L-glutamate, so both of these metabolites are consumed in the biomass reaction*.”

Hence, molecules such as amino acids contained in the genome-scale BSR must be translated into a stoichiometric demand for compounds present in the reduced model. Making this translation manually in a genome-scale model is an error-prone and tedious task and an automated approach as ours supports this step and provides a rigorous approach to obtain a BSR in the compressed network that is consistent with the BSR of the full model. We directly compared the BSRs of *ColiCore* and *ColiPrunedComp* to identify and understand possible sources of different stoichiometries.

Table 2 shows the stoichiometric coefficients of metabolites in the BSR of the *ColiCore* and of the *ColiPrunedComp* model (the BSRs of all models can be found in the Appendix)*.* At a first glance, we can see that almost all metabolites contained in the BSR of *ColiCore* appear also in the BSR of *ColiPrunedComp*, in many cases with comparable amounts.

**Table 2:** Stoichiometries of metabolites in the biomass synthesis reaction of the *ColiCore* and the *ColiPrunedComp* network model. Negative values indicate consumption, positive values production during biomass synthesis

Metabolite	Coefficient in BSR of *ColiCore* [mmol]	Coefficient in BSR of *ColiPrunedComp* [mmol]
2-Oxoglutarate	4.1182	7.4661
3-Phospho-D-glycerate	−1.4960	−1.7175
Acetate		0.5810
Acetyl-CoA	−3.7478	−3.8560
ADP	59.8100	67.7163
AMP		2.2653
ATP	−59.8100	−69.9816
CO2		1.7341
Coenzyme-A	3.7487	4.3809
Dihydroxyacetone-phosphate		−0.1413
Erythrose-4-phosphate	−0.3610	−0.3720
Formate		−0.1080
Fructose-6-phosphate	−0.0709	−0.0945
Fumarate		0.7063
Glucose-6-phosphate	−0.2050	
Glutamate	−4.9414	−6.7339
Glutamine	−0.2557	−1.8075
Glyceraldehyde-3-phosphate	−0.1290	0.0540
Glyoxylate		0.0007
H_c	59.8100	64.5223
H_p		−0.1686
H2O	−59.8100	−57.4196
NAD	−3.5470	−0.3971
NADH	3.5470	0.3971
NADP	13.0279	7.2399
NADPH	−13.0279	−7.2399
NH4		−0.4352
Oxaloacetate	−1.7867	−2.9257
Phosphoenolpyruvate	−0.5191	−0.8102
Pi_c	59.8100	75.3394
Pyruvate	−2.8328	−2.7842
Ribose-5-phosphate	−0.8977	−0.9325
Ribulose-5-phosphate		−0.0398
Succinate		0.8586
Succinyl-CoA		−0.5249
Several external metabolites including trace elements (calcium, sulfate, cobalt, copper, etc.)		(several amounts)

One major difference is that the BSR of *ColiPrunedComp* contains a number of external metabolites, in particular trace elements such as calcium, sulfate, cobalt, copper, magnesium etc. Originally, in the *ColiGS* network, trace elements were taken up by the cell yielding intracellular representatives of these elements which were then consumed by the BSR in the genome-scale model. These essential components of the biomass were kept in the *ColiPrunedComp* model, however, network compression enforced the model to compress the two steps of uptake and consumption of a trace element into one step by integrating the required amount of external trace elements directly in the BSR of *ColiPrunedComp*. Although these external metabolites do not change the feasible network behaviors (and could in principle be removed from the BSR), it is one advantage of our algorithm that these condensed material balances are automatically calculated and still visible in the reduced model. Note that the (cumulative) amount of periplasmatic protons (H_p) consumed in the condensed BSR of *ColiPrunedComp* is related to antiport uptake of trace elements.

Further differences in the BSRs may arise by non-unique ways of representing precursor stoichiometries due to different reference points. For example, *ColiCore* uses glyceraldehyde-3-phosphate (G3P) as a precursor (− 0.129) whereas *ColiPrunedComp* uses instead dihydroxyacetone-phosphate with a similar amount (−0.141). Since both metabolites can be converted into each other by a triose-isomerase reaction, both metabolites can in fact be used as precursors. A similar relationship exists for Ribose-5-phosphate (R5P) and Ribulose-5-phosphate (Ru5P). Ru5P occurs only in the BSR of *ColiPrunedComp* but, due to a simple isomerase reaction between both metabolites, could also be integrated in the value of R5P which is consumed in both BSRs.

Despite those explainable differences, the BSR of *ColiCore* seems to slightly underestimate the demand for most precursors and, to a larger extent, for energy (ATP). On the other hand, the core model consumes a significantly larger amount of NADPH and NADH which, in the final balance and biomass yield, could partially compensate the lower demand for some precursors. As mentioned above, would we add the high efficiency oxidase reaction (9) in the *ColiCore* model, the growth rate (0.9647 h^−1^) would be slightly higher than in the *ColiPrunedComp* model (0.9288 h^−1^). We emphasize that we do not claim that the BSR of *ColiPrunedComp* is necessarily “better” or more realistic than the BSR of the *ColiCore* model in [12]. It is possible that the authors used some specific assumptions in the calculation of the BSR for the *ColiCore* model which may have led to discrepancies with the condensed BSR calculated herein. Nonetheless, the condensed BSR obtained with the *NetworkReducer* algorithm is consistent with the full model and allows for quantitative comparisons. Our algorithm thus facilitates the unbiased and reproducible calculation of condensed BSR stoichiometries in reduced network models from their genome-scale representation.

To further test the quality of *ColiPrunedComp* we exemplarily perfomed flux variability analyses for three different growth scenarios and compared the results with the original genome-scale model (see Additional file 1). We found a very good agreement of fluxes ranges in *ColiPrunedComp* and *ColiGS*. Although the trend of flux ranges in *ColiCore* and *ColiGS* are also similar, several larger differences can be observed.

### Reduction of a cyanobacterial genome-scale model to a module

In a second case study we used a genome-scale model of the phototrophic cyanobacterium *Synechocystis* sp. PCC 6803 and applied *NetworkReducer* to obtain a small metabolic core module which describes CO_2_ fixation by the Calvin-Benson cycle in full detail and allows synthesis of biomass and of a biofuel (ethanol). Such a strongly reduced model may be useful to study basic principles and stoichiometries of, for example, coupled synthesis of biomass and biofuels (see e.g. [13]). The genome-scale model of Knoop et al. [19] (in the variant used by Erdrich et al. [13]) served as starting point. Heterotrophic (night) metabolism was neglected and we again added biomass as internal species and a corresponding pseudo transport reaction for its export. After those minor adaptations the full model contained 599 reactions and 519 internal metabolites (with 96 degrees of freedom; see Additional file 2).

Since the goal was to extract a network module that represents the Calvin cycle and enables maximal phototrophic growth as well as maximal production of ethanol we protected all reactions of the Calvin Cycle, the pathway to ethanol, biomass and ethanol excretion, and light uptake (in total 26 reactions; see Additional file 2). The two protected phenotypes were specified by

$$ \begin{array}{c}{r}_{Photo{n}_{up}}\le 100\\ {}-\mu \le -0.999{\mu}_{max},\end{array} $$

representing maximal phototrophic growth (*μ*_max_ is the maximal growth rate for a photon uptake of 100 mmol/gDW/h), and

$$ \begin{array}{c}{r}_{Photo{n}_{up}}\le 100\\ {}-{r}_{Ethanol}\le -0.999{r}_{\max Ethanol}\end{array} $$

for maximal ethanol production (*r*_maxEthanol_ is the maximal ethanol production rate for a photon uptake of 100 mmol/gDW/h).

As expected and desired, with the focus on a smaller subnetwork, the dimension of the reduced model (pruned and compressed) is much lower than in the *E. coli* core model and contains 37 reactions and 38 internal metabolites. The rank of the stoichiometric matrix is 33 implying four remaining degrees of freedom. The reduced model gives rise to 10 elementary modes reproducing not only the protected functions of pure biomass or ethanol synthesis but also growth-coupled ethanol production (Additional file 2). The condensed biomass synthesis reaction withdraws metabolites (precursors, ATP, NADPH) from the Calvin cycle module needed to build biomass. Since only a subset of “normal” precursors is part of the reduced model (for instance, the reactions and metabolites of the TCA are completely missing) the respective stoichiometric coefficients of precursors in the BSR of the reduced model are relatively large as they are not only needed as direct precursors for the BSR but also as starting point to produce other precursors. Again, *NetworkReducer* ensures that the BSR of the reduced model is consistent with the BSR of the genome scale model therefore generating the same maximal growth rate and biomass yield.

## Conclusions

In this work we presented *NetworkReducer*, a new algorithm for automated reduction of large-scale metabolic network models to obtain meaningful small- or medium-scale models, typically representing the central metabolism or certain modules of interest. The algorithm consists of (i) a pruning step followed by (ii) network compression. Network pruning removes elements of the network that are dispensible for a user-defined set of protected properties and parts. Our algorithm accepts various criteria for specifying protected features. In particular, inequalities as in eqs. (5, 6, 7) provide a high flexibility in defining desired phenotypes. Network pruning is accompanied with a real loss of feasible phenotypes in the model but our algorithm ensures that protected functions and parts are maintained. As a very useful and desired feature, network pruning always delivers a subnetwork of the full system implying that also all phenotypes (feasible flux distributions) of the pruned network are a subset of the feasible behaviors of the full system. As one consequence, for example, all elementary modes of the pruned network are a subset of the elementary modes of the complete system.

Network compression applied after network pruning further condenses the network structure by collapsing some reactions (e.g., from linear pathways) if these are not protected. Network compression works loss-free, that is none of the feasible phenotypes and functions is lost, however, the resulting network is generally not a proper subnetwork of the full system and a mapping of flux distributions (and reactions) between the compressed and the full network can be cumbersome. It will depend on the application whether network compression is applied to the pruned network or not. If, for example, elementary modes are to be calculated in a network, loss-free compression algorithms are commonly used in a preprocessing step [16, 17]. In the *E. coli* example study it was desired to fully condense the network to the predefined core.

Whereas network compression delivers always a unique result, the outcome of the network pruning step can be unique or non-unique, depending on network structure, input parameters, and protected functions and parts. Although reactions are removed in the order of their flux range (smallest range first), reactions with identical flux ranges may exist (e.g., from parallel pathways) from which then one is chosen randomly.

The *E. coli* case study demonstrated the applicability and potential of our approach. We reduced the genome-scale reconstruction *i*AF1260 of Feist et al. [7] to an *E. coli* core model. The reactions and metabolites to be maintained were taken from the core model proposed by Orth et al. [12], which itself was manually derived with *i*AF1260 serving as a basis. With the protected phenotype that the maximal growth rates of *i*AF1260 under aerobic and anaerobic conditions must be achievable also in the condensed model, we used our algorithm to reduce the *i*AF1260 model to its core and compared it with the model of Orth et al. We found a generally good agreement between the manually and the automatically derived model but also some discrepancies. Essentially, two reactions of the *i*AF1260 not contained in the Orth model were retained by *NetworkReducer* to ensure that the maximal growth rates can be achieved. Furthermore, the stoichiometries in the condensed biomass synthesis reactions (BSRs) showed some differences. With our algorithm we can ensure that the condensed BSR fully reflects the stoichiometries of the BSR of the original network thus leading also to the same maximal growth rates and biomass yields. The *E. coli* case study demonstrated the value of our approach for obtaining a fast (less than 5 hours on a typical PC), unbiased, and exact network reduction.

We consider the *E. coli* study as a typical application scenario for our method, namely to reduce a genome-scale network to its core (typically the central metabolism) thereby protecting important properties such as (maximal) biomass or/and product yields. In the general case, a prerequisit is the careful delineation of the central metabolism (to be protected) in the genome-scale network which, however, should be possible for many organisms from prior biological knowledge. Many other reduction problems can be defined as well by using appropriate criteria for protected functions and parts. For example, instead of (or in addition to) the central metabolism, certain modules of a large-scale model (e.g., fatty acid or lipid synthesis) might be maintained in a high resolution while the rest is condensed. The presented case study of extracting a subnetwork of the cyanobacterial metabolism that comprises the Calvin cycle and allows for biomass and biofuel (ethanol) synthesis demonstrated that such an application is also supported by our algorithm. Such extracted subnetworks are also useful for the construction of kinetic models of specific parts of metabolism, while preserving consistency with a genome-scale reconstruction.

An extreme application of *NetworkReducer* would be to compress the full network of an organism to just one single overall reaction consuming external metabolites (substrate, nutrients, etc.) and synthesizing biomass with maximal yield. To simulate this maximal reduction, we again specified maximal aerobic biomass synthesis in *E. coli* as desired phenotype (ATP maintenance demand was not considered) and protected (only) the biomass export reaction. As a result, a “network” with only one overall reaction was maintained which exclusively converts external substrates and nutrients to external products The stoichiometry of this fully condensed BSR with one degree of freedom (*ColiPrunedComp_DOF1)* can be found in the Appendix showing that 10.3893 mmol glucose are required to build 1 gram of biomass in the optimal case.

Although only genome-scale models can provide a complete view on the full functionality of a metabolic network there are several reasons why reduction of large-scale models can be useful or even necessary. First, computationally extensive analyses, such as full enumeration of elementary modes for metabolic pathway analysis, may only be tractable in smaller models. Methods of metabolic flux analysis seeking to calculate internal metabolic fluxes based on measurements of extracellular fluxes or/and on data from isotopic tracer experiments are able to resolve metabolic fluxes for smaller (core) networks only. Smaller models are also useful for didactic or educational purposes [12] and, in fact, might sometimes be more suitable to gain a basic understanding of certain metabolic principles than exploring the “jungle” of a genome-scale network with thousands of reactions and metabolites. Small-scale models are also useful for testing and evaluating new constraint-based analysis methods. Finally, parameter-dependent dynamic modeling of metabolic processes normally focuses on smaller networks and network reduction allows one to cut a network region or module of interest from a genome-scale network which can then be studied by kinetic models. With all these applications in mind, we believe that *NetworkReducer* represents a valuable tool for stoichiometric and constraint-based modeling of metabolic networks.

## Appendix

Biomass synthesis reaction of the genome-scale model *ColiGS*:

0.000223 10fthf + 0.000223 2ohph + 0.5137 ala + 0.000223 amet + 0.2958 arg + 0.2411 asn + 0.2411 asp + 59.984 atp + 0.004737 ca2 + 0.004737 cl + 0.000576 coa + 0.003158 cobalt2 + 0.1335 ctp + 0.003158 cu2 + 0.09158 cys + 0.02617 datp + 0.02702 dctp + 0.02702 dgtp + 0.02617 dttp + 0.000223 fad + 0.007106 fe2 + 0.007106 fe3 + 0.2632 gln + 0.2632 glu + 0.6126 gly + 0.2151 gtp + 54.462 h2o + 0.09474 his + 0.2905 ile + 0.1776 k + 0.01945 kdo2lipid4 + 0.4505 leu + 0.3432 lys + 0.1537 met + 0.007895 mg2 + 0.000223 mlthf + 0.003158 mn2 + 0.003158 mobd + 0.01389 murein5px4p + 0.001831 nad + 0.000447 nadp + 0.011843 nh4 + 0.02233 pe160 + 0.04148 pe160 + 0.02632 pe161 + 0.04889 pe161 + 0.1759 phe + 0.000223 pheme + 0.2211 pro + 0.000223 pydx5p + 0.000223 ribflv + 0.2158 ser + 0.000223 sheme + 0.003948 so4 + 0.000223 thf + 0.000223 thmpp + 0.2537 thr + 0.05684 trp + 0.1379 tyr + 5.5e-05 udcpdp + 0.1441 utp + 0.4232 val + 0.003158 zn2 ➔ **1 gram biomass** + 59.81 adp + 59.81 h + 59.806 pi + 0.7739 ppi.

Biomass synthesis reaction of the *ColiPrunedComp* model ((external) metabolites from the environment compartment have extension _b; from perisplasm: _p):

1.7175 3pg + 3.8560 accoa + 69.9816 atp + 0.1413 dhap + 0.3720 e4p + 0.0945 f6p + 0.1080 for + 1.8075 gln + 6.7339 glu + 57.4196 h2o + 0.1686 h_p + 0.3971 nad + 7.2399 nadph + 0.4352 nh4 + 2.9257 oaa + 0.8102 pep + 0.9613 pi_e + 2.7842 pyr + 0.9325 r5p + 0.0398 ru5p + 0.5249 succoa + 0.0047 ca2_b + 0.0047 cl_b + 0.0032 cobalt2_b + 0.0032 cu2_b + 0.0076 fe2_b + 0.0071 fe3_b + 0.1776 k_b + 0.0079 mg2_b + 0.0032 mn2_b + 0.0032 mobd_b + 0.2503 so4_b + 0.0032 zn2_b ➔ **1 gram biomass** + 0.5810 ac + 67.7163 adp + 7.4661 akg + 2.2653 amp + 1.7341 co2 + 4.3809 coa + 0.7063 fum + 0.0540 g3p + 0.0007 glx + 64.5223 h + 0.3971 nadh + 7.2399 nadp + 75.3394 pi + 0.9613 pi_p + 0.8586 succ + 0.0002 4hba_b + 0.0004 5drib_b + 0.0004 hmfurn_b.

Biomass synthesis reaction of the *ColiCore* model:

1.496 3pg + 3.7478 accoa + 59.81 atp + 0.361 e4p + 0.0709 f6p + 0.129 g3p + 0.205 g6p + 0.2557 gln + 4.9414 glu + 59.81 h2o + 3.547 nad + 13.0279 nadph + 1.7867 oaa + 0.5191 pep + 2.8328 pyr + 0.8977 r5p ➔ **1 gram biomass** + 59.81 adp + 4.1182 akg + 3.7478 coa + 59.81 h + 3.547 nadh + 13.0279 nadp + 59.81 pi.

Biomass synthesis reaction of the *ColiPrunedComp_DOF1* model ((external) metabolites from the environment compartment have extension _b):

10.4352 nh4_b + 10.3893 glc_b + 19.2597 o2_b + 0.9613 pi_b + 0.0047 ca2_b + 0.0047 cl_b + 0.0032 cobalt2_b + 0.0032 cu2_b + 0.0076 fe2_b + 0.0071 fe3_b + 0.1776 k_b + 0.0079 mg2_b + 0.0032 mn2_b + 0.0032 mobd_b + 0.2503 so4_b + 0.0032 zn2_b ➔ **1 gram biomass** + 0.0002 4hba_b + 0.0004 hmfurn_b + 21.3862 co2_b + 47.7272 h2o_b + 9.1844 h_b.

## Additional files

Additional file 1:
**Comparison of flux variabilities in** ***ColiGS,*** ***ColiCore,*** **and** ***ColiPrunedComp*** **for three different growth scenarios.** (XLS 382 kb) Additional file 2:
**Reactions, metabolites, the BSR and elementary modes of the reduced** ***Synechocystis*** **model** ***(SynechocystisPrunedComp).*** (XLS 153 kb)

## Abbreviations

BSR: biomass synthesis reaction
Dof: degree of freedom
FVA: flux variability analysis
FBA: flux balance analysis
